# Point‐of‐care N‐terminal pro B‐type natriuretic peptide assay to screen apparently healthy cats for cardiac disease in general practice

**DOI:** 10.1111/jvim.16156

**Published:** 2021-05-16

**Authors:** Ta‐Li Lu, Etienne Côté, Yu‐Wen Kuo, Hao‐Han Wu, Wen‐Yen Wang, Yong‐Wei Hung

**Affiliations:** ^1^ Cardiospecial Veterinary Hospital Taipei Taiwan, ROC; ^2^ Department of Companion Animals, Atlantic Veterinary College University of Prince Edward Island Charlottetown Prince Edward Island Canada

**Keywords:** biomarker, feline, general practice, heart disease, NT‐proBNP

## Abstract

**Background:**

Point‐of‐care (POC) N‐terminal pro B‐type natriuretic peptide (NT‐proBNP) ELISA test has been evaluated for screening cats for cardiac disease in the referral veterinary setting but less is known about its use in general practice (GP).

**Objectives:**

To evaluate the diagnostic utility of a POC NT‐proBNP ELISA in cats seen in GPs.

**Animals:**

Two hundred and seventeen apparently healthy cats from 21 GPs.

**Methods:**

This was a prospective, cross‐sectional study. Cardiac auscultation and POC NT‐proBNP ELISA were done by veterinarians at their GPs. After enrollment at GPs, cats were sent to a cardiology referral hospital for cardiac auscultation and echocardiographic diagnosis. Results were interpreted based on whether cats had normal or abnormal echocardiographic findings.

**Results:**

Point‐of‐care NT‐proBNP ELISA results differentiated cats in the abnormal group from those in the normal group with a sensitivity of 43%, specificity of 96%. In cats with a heart murmur at GPs, POC NT‐proBNP ELISA results differentiated cats in the abnormal group from those in the normal group with a sensitivity of 71% and a specificity of 92%.

**Conclusion and Clinical Importance:**

In apparently healthy cats in GPs, positive POC NT‐proBNP results are associated with heart disease, warranting an echocardiogram, but negative results do not reliably exclude heart disease. These results suggest POC NT‐proBNP is not an effective screening test for apparently healthy cats in GPs, although its performance is improved if it is used only in cats that have a heart murmur.

**Abbreviations**
ASD: atrial septal defect
AUC: area under the ROC curve
BP: blood pressure
GP: general practice
HCM: hypertrophic cardiomyopathy
HDO: high definition oscillometric
HOCM: hypertrophic obstructive cardiomyopathy
IQR: interquartile range
IVS1‐2D: interventricular septal segment 1 thickness at end‐diastole on 2‐dimensional echocardiography
IVS2‐2D: interventricular septal segment 2 thickness at end‐diastole on 2‐dimensional echocardiography
IVSd‐2D: interventricular septal thickness at end‐diastole on 2‐dimensional echocardiography
IVSd‐M: interventricular septal thickness at end‐diastole on M‐mode echocardiography
LA: left atrium
LA : Ao: left atrial to aortic ratio
LR−: negative likelihood ratio
LR+: positive likelihood ratio
LVFWd‐2D: left ventricular free wall thickness at end‐diastole on 2‐dimensional echocardiography
LVFWd‐M: left ventricular free wall thickness at end‐diastole on M‐mode echocardiography
LVH: left ventricular hypertrophy
LVOT: left ventricular outflow tract
LVWd: maximal left ventricular wall thickness
MS: mitral stenosis
MVD: mitral valve disease
NPV: negative predictive value
NT‐proBNP: N‐terminal pro B‐type natriuretic peptide
POC: point‐of‐care
PPV: positive predictive value
ROC: receiver operating characteristic
SBP: systolic arterial blood pressure
TVD: tricuspid valve dysplasia
UCM: unclassified cardiomyopathy

## 1 INTRODUCTION

The point‐of‐care (POC) N‐terminal pro B‐type natriuretic peptide (NT‐proBNP) ELISA test has the potential to be a convenient tool for screening cats for cardiac disease in general practice (GP). Previous studies evaluated its sensitivity and specificity in cats presented to veterinary teaching hospitals, but the samples studied in these investigations were different from feline samples in GP in important ways. For example, the prevalence of heart disease in past studies of the POC NT‐proBNP ELISA (55%,^1^ 60%^2^) was higher than the prevalence of hypertrophic cardiomyopathy in the general feline sample (approximately 15%).^3^, ^4^ Cats in previous studies underwent POC NT‐proBNP testing when they were suspected of having heart disease, but in the GP setting, veterinarians also might use such testing when they want more information on an apparently healthy cat's cardiac status in the absence of auscultatory abnormalities, such as during a preoperative exam. Furthermore, studies have not systematically evaluated the role of physical examination findings, such as the presence or absence of a heart murmur, when considered in tandem with POC NT‐proBNP ELISA results.

The primary aims of the present study were: (a) to evaluate the diagnostic utility of a POC NT‐proBNP ELISA in a GP cat sample and (b) to evaluate the role of cardiac auscultation when considered in tandem with POC NT‐proBNP ELISA results in the same sample. Secondary aims were: (a) to assess the prevalence of heart murmurs and of echocardiographic abnormalities in apparently healthy cats and (b) to compare the number of cats with cardiac auscultatory abnormalities detected by GP veterinarians with the number of cats with such abnormalities detected by a residency‐trained investigator in a referral veterinary center.

## 2 MATERIALS AND METHODS

### 2.1 Study sample

The study was a prospective, cross‐sectional study approved by the Cardiospecial Veterinary Hospital (“referral hospital,” RVH) Institutional Animal Care and Use Committee (No. 201701). Written, informed owner consent was obtained from the owner of every cat.

Cats that were apparently healthy were recruited prospectively from 21 GPs from April 2017 to January 2019. Cats were considered apparently healthy if they had no clinical signs of illness, no abnormal physical exam findings (other than a heart murmur, gallop sound, arrhythmia, or both gallop sound and arrhythmia) and no history of a medical concern that had required any treatment or diagnostic intervention within 30 days. Exclusion criteria included age <1 year, serum [creatinine] >2.8 mg/dL,^2^, ^5^ systolic arterial blood pressure (SBP) >170 mm Hg, serum total [T4] > 4.0 μg/dL, ongoing medication administration, intolerance to restraint for blood sampling, echocardiography, or both, and >60 days between enrollment at GPs and echocardiography at the RVH. Cardiac auscultatory abnormalities were not an exclusionary criterion.

### 2.2 Experimental protocol

Cats were enrolled when they visited their GPs. The decision to enroll a cat was made by veterinarians at GPs in conjunction with the cats' owner. The stated purpose and benefit of participating in the study was a free heart evaluation at the RVH; no additional incentive was offered. After enrollment, cardiac auscultation was done by veterinarians at GPs. GP veterinarians were asked to identify whether a murmur was present and specifically to choose 1 of the 3 following options: no murmur, systolic murmur, or timing unknown murmur. If a murmur was present, GP veterinarians were asked to note its intensity.^6^ Approximately 3 mL of venous blood was drawn from each cat at GPs. Of this, 1 mL was placed in a heparin tube and centrifuged immediately, for measuring [creatinine] and total [T4] on‐site at GPs (Catalyst One Veterinary Chemistry Analyzer, IDEXX Laboratories, Westbrook, Maine). The remaining 2 mL of blood was placed in an EDTA tube and centrifuged immediately. An aliquot of the EDTA plasma was used to perform the POC NT‐proBNP ELISA test (SNAP Feline proBNP, IDEXX Laboratories, Westbrook, Maine) at GPs on the same day as the cat was enrolled, and the rest of the sample was frozen for subsequent quantitative [NT‐proBNP] measurement (Cardiopet proBNP, IDEXX Laboratories, Westbrook, Maine).^7^ POC NT‐proBNP ELISA testing was performed by trained individuals according to the manufacturer's guidelines and the result was interpreted either by visual inspection or using an automated reading machine (SNAP Pro Analyzer, IDEXX Laboratories, Westbrook, Maine), depending on machine availability in each GP. Visual assay results were considered negative if the color intensity of the sample spot was lighter than the reference spot, or positive if the color intensity of the sample spot was equal to or darker than the reference spot.

After enrolling cats at GPs, veterinarians submitted the signalment information, cardiac auscultation findings, and blood work results of each cat through an online form, and shipped frozen (−20°C) samples consisting of at least 0.5 mL of plasma via freezer truck to the RVH within 2 days (frozen on site at GPs and during transit). After receiving the plasma sample and the cat's baseline information at enrollment from the GP, the RVH contacted the owner and scheduled a cardiac exam date. The plasma sample was stored frozen at −20°C until shipped on dry ice to an external commercial laboratory (IDEXX Reference Laboratories, Westbrook, Maine) for batch measurement of plasma quantitative NT‐proBNP by ELISA.^7^

Upon presentation at the RVH, cats rested in an exam room for ≥5 minutes for acclimation. Then, a high‐definition oscillometric (HDO) blood pressure (BP) monitor (Vet HDO Monitor S + B medVet GmbH, Babenhausen, Germany) was used for performing BP measurements, with the cuff placed around the tail base. The cuff size was selected according to consensus statement guidelines.^8^ If the cat did not tolerate BP measurement, a conventional Doppler BP machine (Ultrasonic Doppler Flow Detector Model 811‐B, PARKS Medical Electronics, Inc, Aloha, Oregon) was used instead. Cardiac auscultation was done by 1 investigator (TL) and heart rate; timing, location, and intensity of murmur, if any; presence/absence of a gallop sound; and presence/absence of an arrhythmia were recorded. Auscultation results were classified as abnormal if a heart murmur, gallop sound, arrhythmia, or both gallop sound and arrhythmia, was/were present, and normal if none of these abnormalities was noted. All cats underwent routine 2‐dimensional (2D), M‐mode, and Doppler echocardiographic examinations,^9^ unsedated, while gently restrained in lateral recumbency. Echocardiograms were performed by 1 investigator (TL) using an ultrasound unit (CX 50, Philips Ultrasound, Bothell, Washington) equipped with a 12‐4 MHz phased‐array transducer and with simultaneous electrocardiographic monitoring. End‐diastolic left ventricular wall thickness measurements were performed using both M‐mode and 2‐dimensional echocardiography on the right parasternal short‐axis view at the level of the papillary muscles using a leading‐edge‐to‐leading‐edge technique.^10^ In M‐mode, the left ventricular free wall (LVFWd‐M) and the interventricular septum (IVSd‐M) were measured at end‐diastole. In 2D, 4 segments were measured on short axis at end‐diastole (IVSd‐2D, LVFWd‐2D, IVS1‐2D, and IVS2‐2D) as previously described.^3^ The maximal diastolic left ventricular wall thickness measurement among the IVSd‐M, LVFWd‐M, IVSd‐2D, LVFWd‐2D, IVS1‐2D, and IVS2‐2D was identified as the maximum LVWd for later analysis. Focal left ventricular hypertrophy (LVH), which was identified via observation of long‐axis and additional short‐axis 2D echocardiographic images beyond those already described, was confirmed via measurement of wall thickness on such images and was recorded. The left atrial to aortic ratio (LA : Ao) was calculated from measurements made on the right parasternal short‐axis view images at the end of ventricular systole.^11^ Abnormal cats were classified as having hypertrophic cardiomyopathy (HCM); hypertrophic obstructive cardiomyopathy (HOCM); unclassified cardiomyopathy (UCM); or noncardiomyopathic heart disease, which included valvular disease, congenital heart malformations, and primary arrhythmia without echocardiographic abnormalities. HCM was defined as regional or generalized LVH where diastolic left ventricular wall thickness (maximum LVWd) exceeded 6 mm. HOCM was defined as HCM with left ventricular outflow tract (LVOT) obstruction as inferred by peak LVOT velocity >2 m/s in the absence of a visible abnormality of the aortic valve. The degree of LVH was combined with LA : Ao measurements to create 3 categories of severity for HCM and HOCM using a scale modified from a previous study^12^: mild (maximum LVWd >6.0‐6.5 mm and LA : Ao ≤1.5), moderate (maximum LVWd >6.5‐7.0 mm and LA : Ao ≤1.8 or maximum LVWd >6.0‐6.5 mm and LA : Ao >1.5‐1.8), and severe (maximum LVWd >7.0 mm with any LA/Ao or maximum LVWd >6.0 mm and LA : Ao >1.8). Cats with maximum LVWd >5.5‐6.0 mm and LA : Ao ≤1.5 were considered to be equivocal for HCM/HOCM. UCM was diagnosed when there was LA : Ao >1.6, maximum LVWd <6.0 mm, LV fractional shortening >20%, and no intracardiac shunts or stenoses could be identified. The diagnosis was made and disease severity was scored by the same investigator (TL). In cases where determination, analysis, or both, of echocardiographic findings was/were challenging, a second investigator (YH) reviewed the case and the final result was determined by agreement between the 2 investigators. All investigators were blinded to POC NT‐proBNP ELISA results until echocardiographic results had been tabulated and related calculations had been completed.

### 2.3 Statistical analysis

Software (IBM SPSS Statistics for Windows, Version 21.0. IBM Corp. Armonk, New York) was used for statistical analysis. Continuous data are expressed as median and interquartile range (IQR). Cats were analyzed in 2 groups (normal, abnormal). For the purpose of these analyses, the normal group consisted of cats that had normal and equivocal echocardiographic findings^13^ and the abnormal group consisted of cats that had abnormal echocardiographic findings. Age, body weight, heart rate, blood pressure, serum [creatinine], serum total [T4], plasma [NT‐proBNP] and echocardiographic measurements (IVSd‐M, LVFWd‐M, IVSd‐2D, LVFWd‐2D, IVS1‐2D, IVS2‐2D, maximum LVWd, LA : Ao) were compared between normal and abnormal groups using Mann‐Whitney tests. Differences in sex and neuter status between normal and abnormal groups were compared using the Fisher's exact test. Quantitative NT‐proBNP ELISA results were compared to positive/negative POC NT‐proBNP ELISA results and to normal/abnormal groups using Mann‐Whitney tests. The utility of POC NT‐proBNP ELISA and quantitative NT‐proBNP ELISA for differentiating the normal group from the abnormal group was evaluated by calculating sensitivity, specificity, positive likelihood ratio (LR+), negative likelihood ratio (LR−), positive predictive value (PPV), negative predictive value (NPV) and accuracy. Receiver operating characteristic (ROC) curves were constructed and the area under each ROC curve (AUC) was calculated to assess the performance of the POC NT‐proBNP ELISA and quantitative NT‐proBNP ELISA for differentiating between normal and abnormal groups. A kappa value was calculated to evaluate the agreement of auscultation results between GP veterinarians and the resident‐trained clinician. For echocardiographic diagnoses with fewer than 10 cats, only descriptive statistics are presented. Statistical significance was defined as *P* < .05.

## 3 RESULTS

**FIGURE 1:**
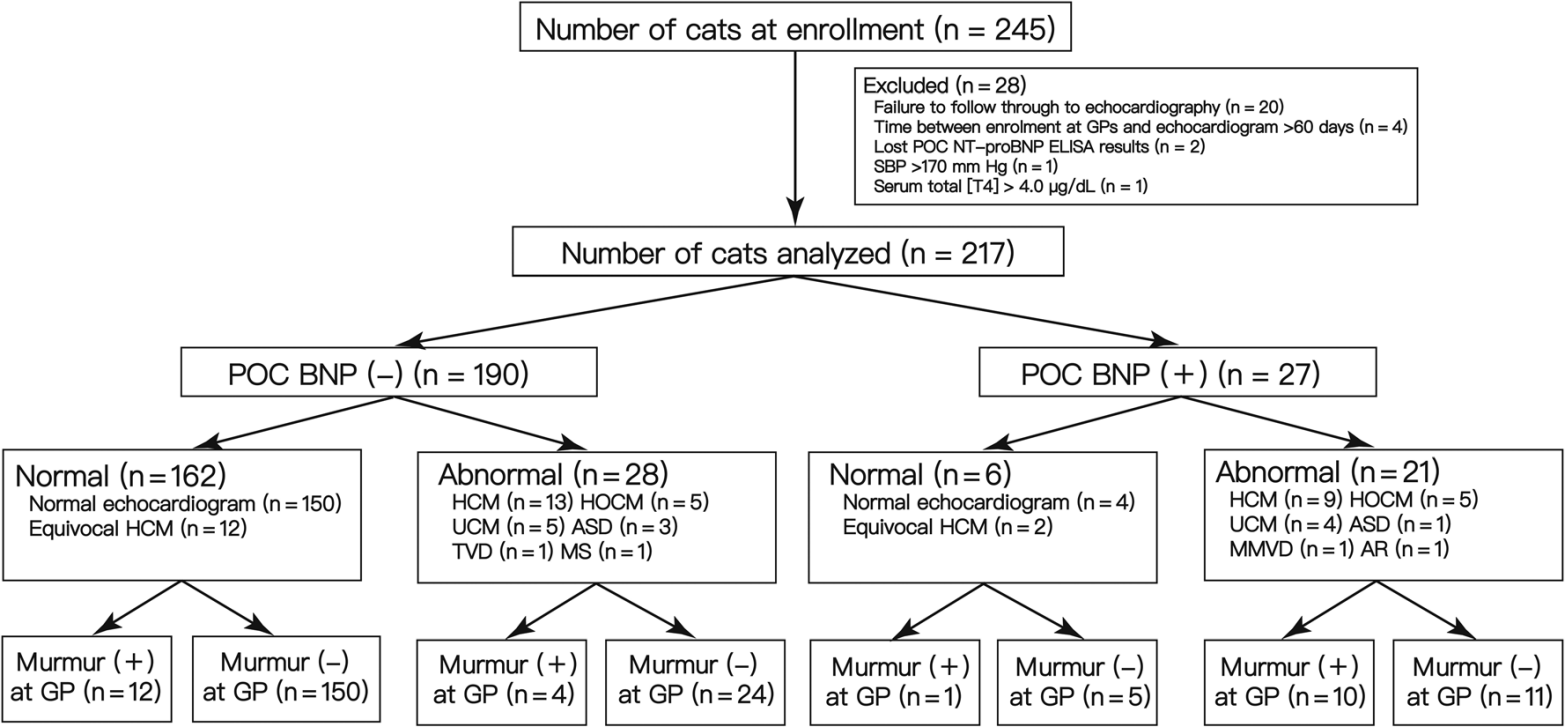
Flow chart of cat enrollment and diagnostic results. ASD, atrial septal defect; GP, general practice; HCM, hypertrophic cardiomyopathy; HOCM, hypertrophic obstructive cardiomyopathy; MS, mitral stenosis; MMVD, myxomatous mitral valve disease; SBP, systemic arterial blood pressure; TT4, serum total thyroxine concentration; TVD, tricuspid valve dysplasia; UCM, unclassified cardiomyopathy

Two hundred and forty‐five cats initially were enrolled by GP veterinarians. Twenty‐eight cats were excluded due to failure to follow through to echocardiography (n = 20), time between enrollment at GPs and echocardiogram >60 days (n = 4), lost POC NT‐proBNP ELISA results (n = 2), and SBP >170 mm Hg and serum total [T4] > 4.0 μg/dL (n = 1 each), leaving 217 cats for final analysis (Figure 1). There were 7 intact males, 119 castrated males, 10 intact females, and 81 spayed females. The median age was 8 years (IQR, 4‐11 years). The median weight was 5 kg (IQR, 4‐6 kg). Breeds were domestic shorthair (n = 96); purebred crosses (n = 37); Chinchilla (n = 19); Persian (n = 16); American shorthair (n = 15); Scottish fold (n = 13); Maine coon, English shorthair, and Russian blue (n = 3 each); Ragdoll, Abyssinian, Munchkin, and Himalayan (n = 2 each); and Siamese, Sphynx, Exotic shorthair, and Norwegian forest (n = 1 each).

In this sample of 217 cats without overtly apparent physical signs of heart disease, 49 (23%) had abnormal echocardiographic findings, consisting of 22/49 (45%) HCM, 10/49 (20%) HOCM, 9/49 (21%) UCM, and 8/49 (16%) noncardiomyopathy‐type cardiac disease (atrial septal defect (ASD, n = 4) and aortic regurgitation, myxomatous mitral valve disease (MMVD), tricuspid valve dysplasia (TVD), mitral stenosis (MS), and 3rd degree atrioventricular block (n = 1 each; 1 cat had MMVD + 3rd degree atrioventricular block)). Cats with UCM had Doppler LV inflow profiles that precluded a diagnosis of restrictive cardiomyopathy: E‐A fusion (n = 4), E/A < 2 (n = 4), and E/A > 2 with heart rate 91 beats/min due to 2nd degree AV block (n = 1). Fourteen cats had echocardiographic findings that were equivocal for HCM. In the 32 cats with HCM/HOCM, echocardiographic abnormalities were mild (8/32, 25%), moderate (10/32, 31%), or severe (14/32, 44%). In 2 cats, HCM was diagnosed subjectively (1 papillary muscle hypertrophy, 1 LVOT focal hypertrophy; both mild). There were 5 challenging cases, and the diagnosis that was reached by agreement was 4 normal echocardiographic findings and 1 HCM.

The normal group consisted of 168 cats without echocardiographic abnormalities and the abnormal group consisted of 49 cats. There were no significant differences between normal and abnormal groups in terms of sex (*P* = .63), neuter status (*P* = .55), body weight (*P* = .46), heart rate (*P* = .38), SBP (*P* = .11), serum [creatinine] (*P* = .78) or serum total [T4] (*P* = .6) (Table 1). In contrast, there was a significant difference between the 2 groups in terms of age (*P* = .04), IVSd‐M (*P* < .001), LVFWd‐M (*P* < .001), IVSd‐2D (*P* = .002), LVFWd‐2D (*P* = .02), IVS1‐2D (*P* < .0001), IVS2‐2D (*P* = .02), maximum LVWd (*P* < .001), and LA : Ao (*P* = .002) (Table 1).

**TABLE 1:** Characteristics of the normal and abnormal groups at the time of evaluation at the RVH

	Normal group	Abnormal group				*P*‐value (between normal and abnormal group)	95% confidence interval
		Overall	HCM/HOCM	UCM	Other		
Age (y)	7 (4‐11)	9 (7‐11)*	9 (7‐11)	11 (10‐12)*	9 (4‐11)	.04	0‐3
Sex (M/F) (%)	96/72 (57%/43%)	30/19 (61%/39%)	20/12 (63%/38%)	3/6 (33%/67%)	7/1 (87%/13%)	.63	
Neutered (Y/N) (%)	156/12 (93%/7%)	44/5 (90%/10%)	29/3 (91%/9%)	9/0 (100%/0%)	6/2 (75%/25%)	.55	
BW (kg)	5 (4‐6)	5 (4‐6)	5 (4‐6)	6 (5‐7)*	5 (4‐5)	.46	0‐1
HR (bpm)	198 (175‐220)	186 (162‐216)	195 (180‐228)	195 (171‐212)	150 (138‐117)*	.38	−18‐6
SBP (mm Hg)	139 (131‐151)	146 (136‐154)	144 (137‐155)	150 (142‐155)	146 (128‐152)	.11	−1‐10
Creatinine (mg/dL)	1.8 (1.6‐2.1)	1.9 (1.5‐2.1)	2 (1.6‐2.1)	1.8 (1.6‐2.1)	2 (1.5‐2.4)	.78	−0.1‐0.2
TT4 (μg/dL)	2.4 (2.1‐2.8)	2.4 (2.2‐2.8)	2.4 (2.3‐2.8)	2.5 (2.4‐2.7)	2.4 (1.9‐3.2)	.6	−0.1‐0.2
IVSd‐M (mm)	4.39 (3.82‐4.96)	6.15 (4.38‐6.63)*	6.49 (6.19‐6.94)*	4.08 (3.36‐4.88)	4.34 (3.47‐4.82)	<.001	0.96‐1.78
LVFWd‐M (mm)	4.27 (3.88‐4.93)	5.1 (4.25‐6.57)*	5.52 (5.003‐6.87)*	4.59 (4.07‐4.78)	4.18 (3.87‐5.22)	<.001	0.43‐1.16
IVSd‐2D (mm)	4.15 (3.53‐4.65)	5.27 (4.5‐6.85)*	6.2 (5.51‐6.94)*	4.46 (4.1‐5.03)	4.35 (4.08‐4.8)	.002	0.039‐0.209
LVFWd‐2D (mm)	4.24 (3.86‐4.79)	5.45 (4.1‐6.06)*	5.96 (5.23‐6.08)*	4.87 (4.1‐5.47)	4.25 (3.95‐5.15)	.02	0.021‐0.162
IVS1‐2D (mm)	4.53 (4.03‐4.99)	6.24 (4.46‐6.75)*	6.66 (6.17‐6.86)*	4.31 (3.91‐5.09)	4.75 (4.09‐4.97)	<.0001	0.103‐0.189
IVS2‐2D (mm)	4.14 (3.39‐4.77)	5.12 (3.75‐5.81)*	5.59 (5.03‐6.41)*	4.48 (3.52‐5.22)	4.64 (4.13‐5.02)	.02	0.17‐0.172
Maximum LVWd (mm)	4.84 (4.39‐5.27)	6.39 (5.27‐7.07)*	6.76 (6.42‐7.93)*	4.95 (4.2‐5.55)	5.02 (4.28‐5.23)	<.001	1.12‐1.82
LA/Ao	1.41 (1.27‐1.51)	1.53 (1.31‐1.78)*	1.5 (1.3‐1.72)	1.82 (1.72‐1.84)*	1.33 (1‐1.7)	.002	0.07‐0.25

*Notes*: Continuous variables are reported as median (interquartile range). Categorical variables are reported as number (%).

Abbreviations: bpm, beats per minute; BW, body weight; HCM, hypertrophic cardiomyopathy; HOCM, hypertrophic obstructive cardiomyopathy; HR, heart rate; IVS1‐2D, interventricular septal segment 1 thickness at end‐diastole on 2‐dimensional echocardiography; IVS2‐2D, interventricular septal segment 2 thickness at end‐diastole on 2‐dimensional echocardiography; IVSd‐2D, echocardiographic two‐dimensionally‐derived echocardiographic interventricular thickness in diastole; IVSd‐M, echocardiographic M‐mode‐derived interventricular thickness in diastole; LA : Ao, left‐atrial‐to‐aortic ratio; LVFWd‐2D, echocardiographic two‐dimensionally‐derived left ventricular free wall thickness; LVFWd‐M, echocardiographic M‐mode‐derived left ventricular free wall thickness; maximum LVWd, maximal diastolic left ventricular wall thickness measurement among IVSd‐M, LVFWd‐M, IVSd‐2D, LVFWd‐2D, IVS1‐2D, and IVS2‐2D; SBP, systemic arterial blood pressure; TT4, serum total thyroxine concentration; UCM, unclassified cardiomyopathy.

* *P* < .05 (compared to normal group).

POC NT‐proBNP ELISA results were interpreted by visual inspection in 199/217 cats and by an automated reading machine in 112/217 cats; that is, 94 cats had results interpreted using both methods. There were 0/94 discordant results.

**FIGURE 2:**
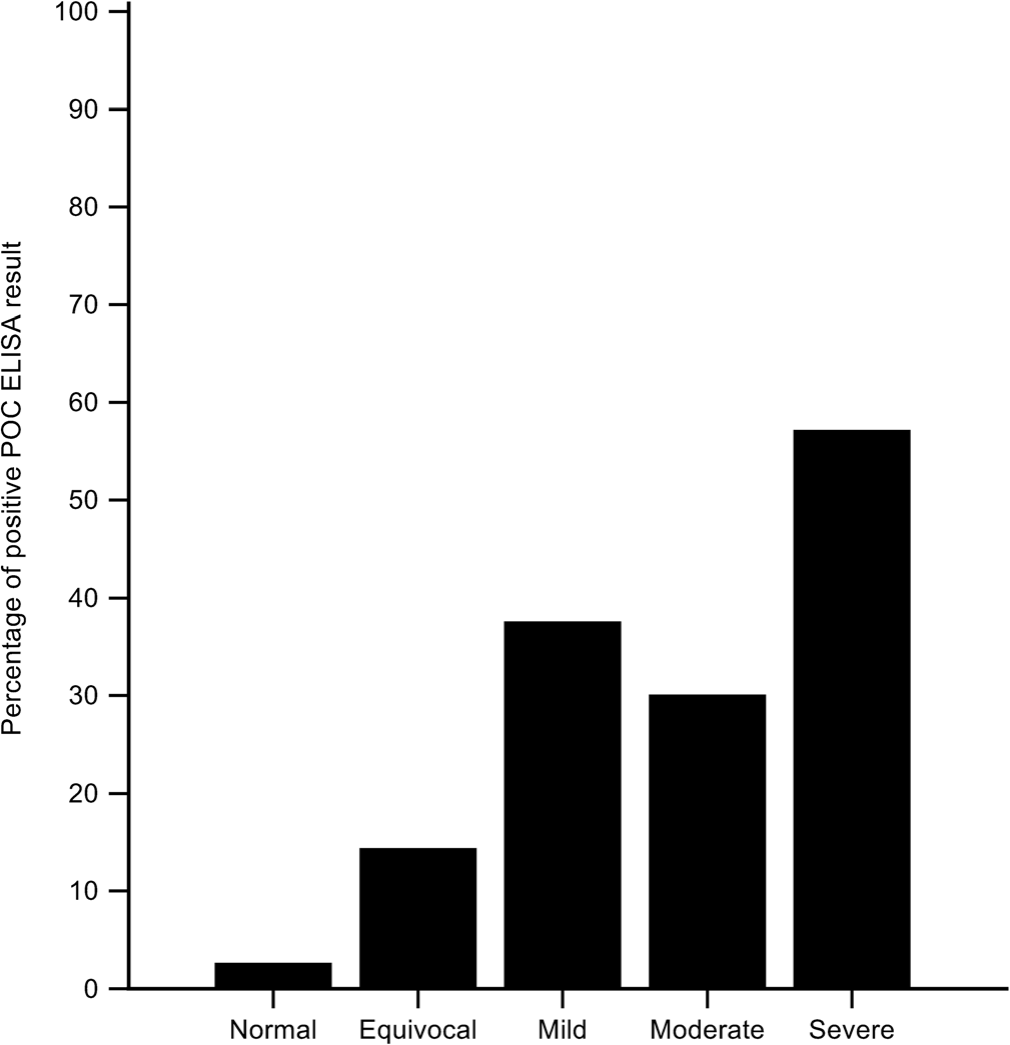
Percentage of positive POC ELISA results in cats with a normal echocardiogram and cats with equivocal, mild, moderate, and severe echocardiographic changes consistent with HCM/HOCM

Twenty‐seven of the 217 POC NT‐proBNP ELISA results (12%) were positive: 4/154 (3%) in normal cats, 2/14 (14%) in cats with echocardiographic results that were equivocal for HCM/HOCM, 3/8 (38%) in mildly affected HCM/HOCM cats, 3/10 (30%) in moderately affected HCM/HOCM cats, and 8/14 (57%) in severely affected HCM/HOCM cats (Figure 2).

POC NT‐proBNP ELISA results differentiated cats in the abnormal group from those in the normal group with a sensitivity of 43%, specificity of 96%, AUC of 0.7, LR+ of 12, LR− of 0.59, PPV of 78% and NPV of 85%. No significant differences were noted when analyses were performed with equivocal cases in the abnormal group.

**FIGURE 3:**
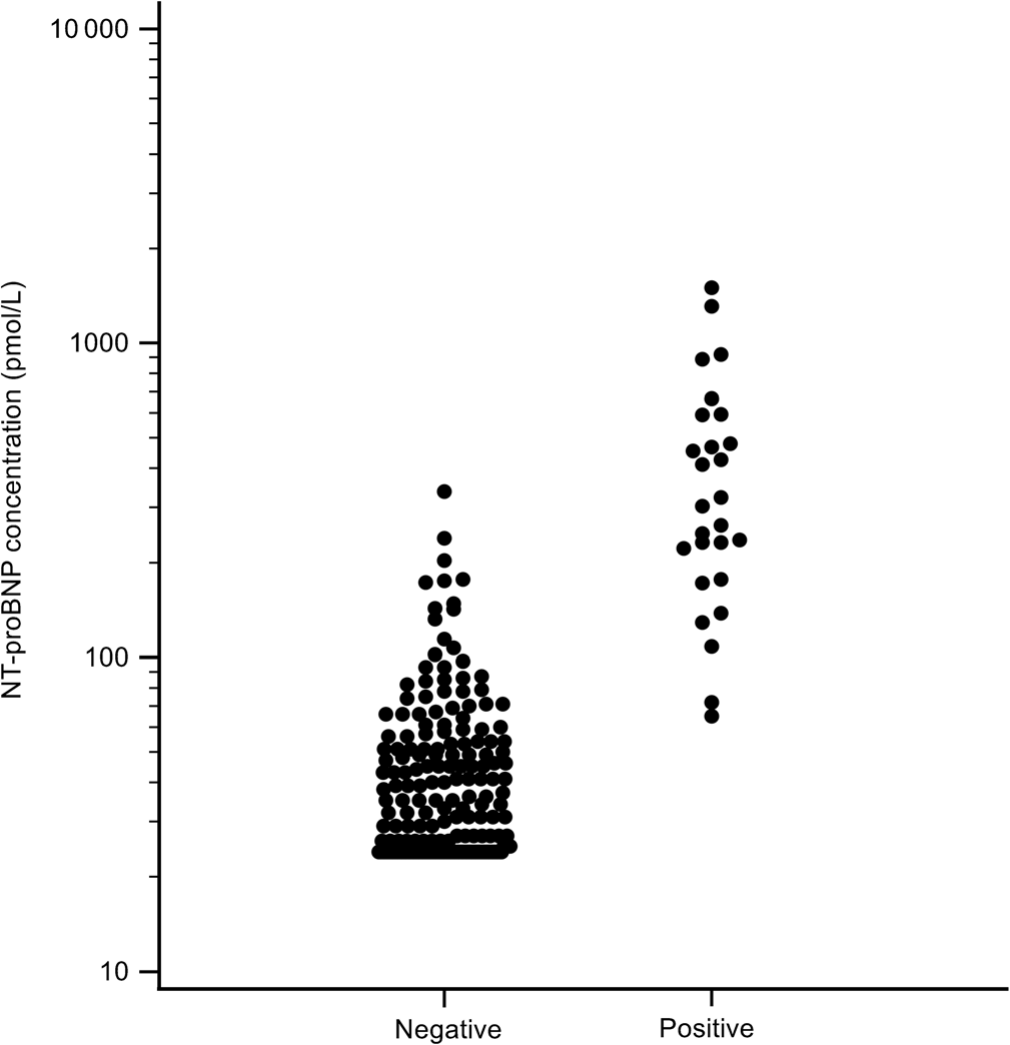
Quantitative NT‐proBNP concentrations in cats with either a positive or a negative POC NT‐proBNP ELISA result. Note the use of a log scale on the y‐axis

**FIGURE 4:**
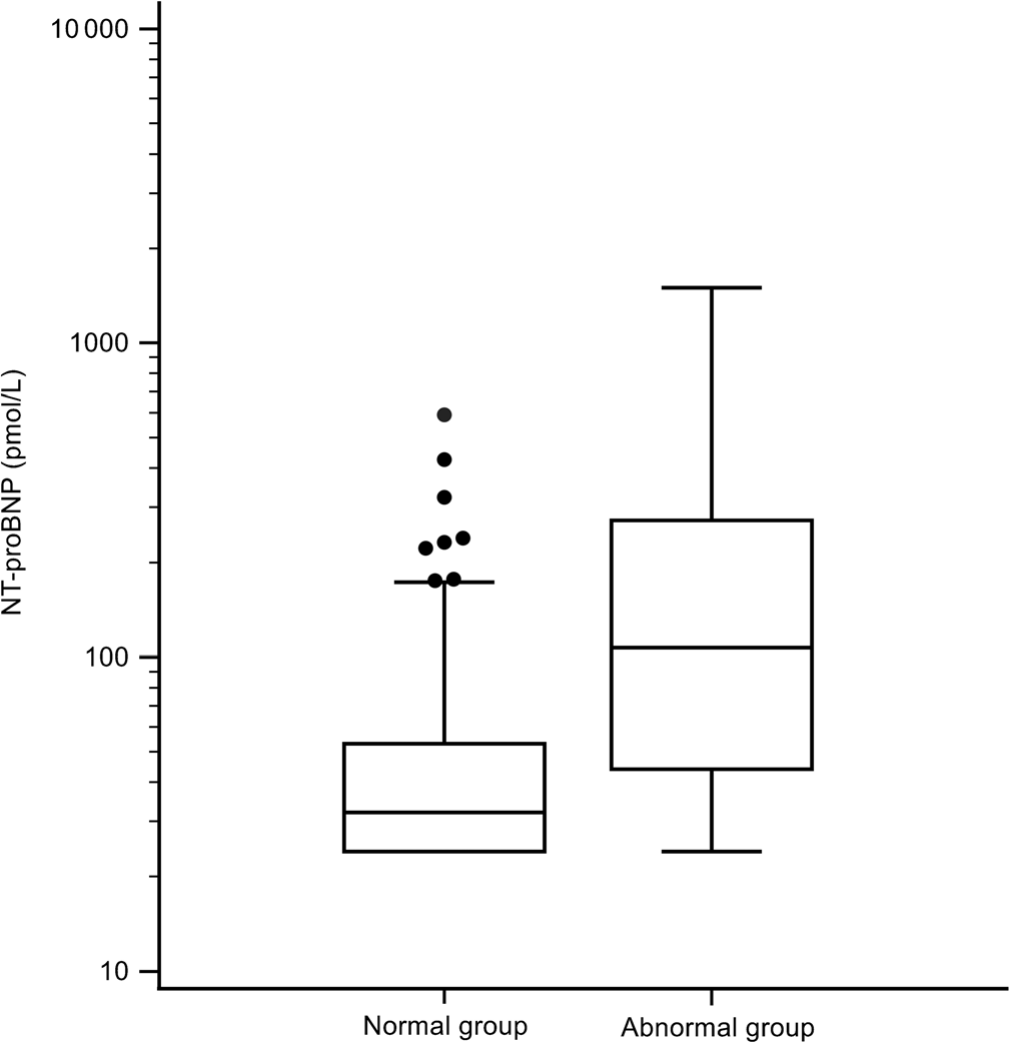
Box and whisker plot of NT‐proBNP concentrations in the normal group and the abnormal group. Note the use of a log scale on the y‐axis

The median plasma [NT‐proBNP] in cats with a positive POC ELISA NT‐proBNP was 303 pmol/L (IQR, 188.3‐563.8 pmol/L), which was significantly higher than the median plasma [NT‐proBNP] of cats with a negative POC ELISA NT‐proBNP result (33.5 pmol/L; IQR, 24‐53 pmol/L), *P* < .001) (Figure 3). The median plasma [NT‐proBNP] was 32 pmol/L (IQR, 24‐53 pmol/L) in the normal group, which was significantly lower than the median value for the abnormal group (107 pmol/L; IQR, 44‐273 pmol/L) (*P* < .0001) (Figure 4).

Using a cutoff value of 99 pmol/L^13^ conferred a sensitivity of 51%, a specificity of 92% and an AUC of 0.72 to the quantitative NT‐proBNP ELISA measurement in the present study (Table 2). A cutoff value of 46 pmol/L^13^ separated normal from abnormal groups with a sensitivity of 74% and a specificity of 67%, and a cutoff value of 85 pmol/L had a sensitivity of 59% and a specificity of 91%.

**TABLE 2:** Performance of POC and quantitative (cutoff value of 99 pmol/L) NT‐proBNP ELISAs to differentiate cats in the abnormal group from those in the normal group

	POC ELISA	95% confidence interval	Quantitative ELISA	95% confidence interval
Sensitivity	43%	29‐58%	51%	36‐66%
Specificity	96%	92‐99%	92%	87‐96%
AUC	0.7	0.63‐0.76	0.72	0.65‐0.78
Positive likelihood ratio	12	5.1‐28	6.59	3.7‐11.9
Negative likelihood ratio	0.59	0.5‐0.8	0.53	0.4‐0.7
Positive predictive value	78%	60‐89%	66%	52‐78%
Negative predictive value	85%	82‐88%	87%	83‐90%
Accuracy	84%	79‐89%	83%	77‐88%

Abbreviations: AUC, area under the receiver operating characteristic curve; POC, point‐of‐care NT‐proBNP.

There were 28 cats in the abnormal group of 49 cats (57%) that had a negative POC NT‐proBNP ELISA result, representing false‐negative results. These consisted of 13 HCM (4 mild, 6 moderate, 3 severe), 5 HOCM (1 mild, 1 moderate, 3 severe), and 10 other cardiac diseases. The median plasma quantitative [NT‐proBNP] of these 28 cats was 47 pmol/L (IQR, 29‐90 pmol/L), and 5 had an NT‐proBNP concentration >100 pmol/L (107 pmol/L, 142 pmol/L, 148 pmol/L, 203 pmol/L, 337 pmol/L).

There were 6 cats in the normal group that had a positive POC NT‐proBNP ELISA result, representing false‐positive results. None had identifiable systemic abnormalities that could explain this result, other than 1 cat with serum [creatinine] that was slightly greater than the instrument's reference interval for cats (0.8‐2.4 mg/dL). These 6 cats were of 5 breeds (DSH [n = 2], American shorthair, Chinchilla, Persian, Purebred crosses [n = 1 each]), were 7‐15 years old, and had a serum [creatinine] = 1.8‐2.6 mg/dL, serum total [T4] = 1.4‐3.7 μg/dL, SBP = 111‐167 mm Hg, and plasma [NT‐ proBNP] = 72‐592 pmol/L.

Upon presentation at GPs, 27 cats (27/217, 12%) had abnormal cardiac auscultation findings: 9 cats had a systolic heart murmur and 18 cats had a heart murmur of unknown timing. There were 1 grade 1/6, 6 grade 2/6, 10 grade 3/6 and 10 intensity‐unknown murmurs ausculted by GP veterinarians. Echocardiographic results placed 13/27 (48%) GP murmur cats in the normal group and 14/27 (52%) GP murmur cats in the abnormal group. Upon subsequent presentation at the RVH for echocardiography, 50 cats had abnormal cardiac auscultation findings (47 systolic murmurs, 3 gallops). There were 13 grade 2/6 murmurs, 6 grade 3/6 murmurs, and 28 grade 4/6 murmurs. Echocardiographic results placed 26/50 (52%) cats in the normal group and 24/50 (48%) in the abnormal group. Comparing auscultation findings between GP veterinarians and the RVH investigator (TL), the kappa value was 0.24. There were 14 cases where both a GP veterinarian and the investigator (TL) heard a murmur. Those 14 cats had a loud heart murmur (grade 3/6 (n = 1), grade 4/6 (n = 13)). There were 36 cats in which the investigator (TL) heard either a murmur (n = 33; grade 2/6 (n = 14), grade 3/6 (n = 5), grade 4/6 (n = 14)) or a gallop (n = 3) but the GP veterinarian did not. There were 13 cats in which the GP veterinarian had ausculted a murmur, but the investigator (TL) did not. In all 13 cats, the GP veterinarian indicated that he or she was not sure about murmur timing and intensity.

Of 27 cats with a heart murmur at GPs, 11 had a positive POC result and 16 had a negative POC result. Ten of 11 cats (91%) with a heart murmur and POC‐positive result had an abnormal echocardiogram (5 HCM [1 mild, 2 moderate, 2 severe], 2 HOCM [both severe], 3 other), compared to 4/16 cats (25%) with a heart murmur and POC‐negative result (2 HCM [1 mild, 1 severe], 2 HOCM [both severe]) (*P* = .002). In cats with a heart murmurs at GPs, POC NT‐proBNP ELISA results differentiated cats in the abnormal group from those in the normal group with a sensitivity of 71%, specificity of 92%, LR+ of 9.29, LR− of 0.31, PPV of 91% and NPV of 75%. In cats with normal cardiac auscultation findings at GPs, the sensitivity and specificity of POC NT‐proBNP ELISA for identifying that a cat was in the abnormal group were 31% and 97%, respectively. The LR+ was 9.74, the LR− was 0.71, the PPV was 69% and the NPV was 87%. Similar findings were also noted when comparing POC NT‐proBNP ELISA results between cats with normal auscultation findings and cats with abnormal auscultation findings at the RVH (Table 3).

**TABLE 3:** Utility of POC NT‐proBNP ELISA test to detect echocardiographic abnormalities of cats from different auscultation result groups, at both general practices and the RVH

	Abnormal auscultation at GP	Normal auscultation at GP	Abnormal auscultation at RVH	Normal auscultation at RVH	All cats
Number of cats	27	190	50	167	217
Abnormal echocardiogram prevalence	52%	18%	48%	15%	23%
Sensitivity	71%	31%	58%	28%	43%
Specificity	92%	97%	85%	99%	96%
AUC	0.82	0.64	0.72	0.63	0.7
Positive likelihood ratio	9.29	9.74	3.79	19.88	12
Negative likelihood ratio	0.31	0.71	0.49	0.73	0.59
Positive predictive value	91%	69%	78%	78%	78%
Negative predictive value	75%	87%	69%	89%	85%
Accuracy	81%	85%	72%	88%	84%

Abbreviations: AUC, area under the receiver operating characteristic curve; GP, general practice; RVH, referral veterinary hospital.

## 4 DISCUSSION

The results of the present study suggest that POC NT‐proBNP is not an effective screening test when applied to cats regardless of auscultation results; however, its sensitivity increases when it is used for assessing cats that have a heart murmur. This finding emphasizes the value of cardiac auscultation before performing the POC NT‐proBNP ELISA test, because performing the POC NT‐proBNP ELISA test on a cat with a heart murmur is more likely to produce diagnostically useful information than performing it on a cat without a heart murmur. Even so, the combination of ausculting a heart murmur and obtaining a positive POC NT‐proBNP test result would be expected to detect heart disease in a cat with no overt clinical cardiovascular signs in approximately 7 out of 10 cases. These results signify that using POC NT‐proBNP testing in cats without heart murmurs seen in GPs is unlikely to help GP veterinarians distinguish between cats with heart disease and cats without heart disease. When a POC NT‐proBNP result is positive, however, it is highly likely that the cat has echocardiographically‐identifiable heart disease, which can be of a mild, moderate, or severe degree.

The present results (sensitivity: 43%; specificity: 96%) indicated that POC NT‐proBNP ELISA had lower sensitivity and higher specificity for detection of heart disease in apparently healthy cats when compared to 1 earlier study (sensitivity: 84%; specificity: 83%)^2^ and similar results compared to another study (sensitivity: 65%; specificity: 100%).^1^ The differences between our results and those of previous studies could be due to differences in cat selection. Previous studies^1^, ^2^ were performed in teaching hospital settings where cats could have been referred due to a suspicion of heart disease. In order to reflect the clinical scenario of how veterinarians use POC NT‐proBNP ELISA in GPs in our region, we designed this study to recruit cats directly at GPs and we asked veterinarians to perform on‐site POC NT‐proBNP ELISA testing. Thus, we did not select for cats with suspected heart disease. Another possible explanation for the differences between the present results and past results from similar studies could be the selection criteria for normal and abnormal groups. In 1 earlier study, the normal group included cats with echocardiographically normal hearts, equivocal changes, and evidence of mild heart disease, and the abnormal group included cats with echocardiographic evidence of moderate and severe heart disease.^2^ In another study, there was no evaluation of disease severity and the normal group consisted of cats with normal echocardiographic findings, whereas the abnormal group included cats with all types of echocardiographic abnormalities.^1^ In the present study, in order to reduce misdiagnoses in cases with left ventricular wall measurements that were neither convincingly normal nor convincingly abnormal, we described these cases as equivocal and included them in the normal group for analysis. Despite these differences in study design and cohort composition, our results were similar to those of 1 of the studies.^1^

For feline occult cardiac disease, echocardiography currently is the gold‐standard diagnostic tool,^14^ but high cost and limited availability could make it inaccessible to some cat owners. The high specificity and positive predictive value of POC NT‐proBNP ELISA shown in the present study mean that a positive result can make GP veterinarians be more confident in identifying heart disease and referring the cat for echocardiography. However, the low sensitivity of POC NT‐proBNP ELISA and low percentage of positive POC NT‐proBNP ELISA results in HCM/HOCM cats (Figure 1) indicate that the ability of POC NT‐proBNP ELISA to correctly identify cats with heart disease in this cohort of cats is low. Our results support the role of POC NT‐proBNP ELISA testing when GP veterinarians want support for the decision to refer a cat for echocardiography, and not as a screening test to rule out occult heart disease in cats.

There were 28 cats with negative POC NT‐proBNP ELISA results in the abnormal group (false negative cases). Five of them had quantitative plasma [NT‐proBNP] >100 pmol/L (107, 142, 148, 203, 337); 4 of these values were in the POC's transition zone from negative to positive. Possible explanations for POC NT‐proBNP ELISA false negative results include echocardiographic abnormalities that were caused by transient myocardial thickening,^15^ limitations of echocardiographic interpretation caused by intraobserver variability^16^ and differences in RAAS and BNP activation among different cats. Test inaccuracy is possible, although the low median quantitative [NT‐proBNP] of 47 pmol/L in these cats suggests that the POC test effectively measures [NT‐proBNP] and that the inconsistency is not specific to the POC test, but rather is between echocardiographic results and [NT‐proBNP], whether POC or quantitative.

There were 6 false positive cases. One of these had NT‐proBNP <100 pmol/L (72 pmol/L), which can be explained by POC ELISA test performance variability or its accuracy when applied to this cohort of apparently healthy cats. The other 5 cats had high NT‐proBNP concentration of 222, 232, 232, 426 and 592 pmol/L, respectively, indicating that the POC ELISA test accurately reflected circulating [NT‐proBNP]. Since we ruled out serum [creatinine] >2.8 mg/dL, SBP > 170 mm Hg, and serum total [T4] > 4.0 μg/dL in our cats, the reason for a high plasma [NT‐proBNP] in these cats with a normal echocardiogram is not determined from this study. Whether measurement of plasma [NT‐proBNP] can detect cardiac disease before structural heart disease is noted echocardiographically in some cats is a question that deserves further investigation based on the results of the present study.

The prevalence of heart murmurs in overtly healthy cats, and correlation to echocardiographic findings, has been studied in referral veterinary hospitals^3^, ^17^, ^18^, ^19^, ^20^ and rehoming shelters.^4^ The findings of this study provide insights on the existence of heart murmurs and structural heart disease in apparently healthy cats presented to GPs for veterinary care. These findings regarding the prevalence of heart murmurs are comparable to those described in some studies elsewhere in the world^3^, ^17^ and not others.^4^ Differences also exist in the prevalence of echocardiographically‐demonstrated LVH, which has been identified in 14.7% and 15% of apparently healthy cats in previous studies^3^, ^4^ compared with 23% (49/217) in the present study. A possible explanation for a higher prevalence of heart disease in the present study could be the increased prevalence of cardiomyopathy in older cats, as noted previously,^4^ since cats in the abnormal group in the present study were significantly older than cats in the normal group.

The identification of heart murmurs by specialists examining apparently healthy cats is well‐described.^3^, ^4^, ^17^, ^18^, ^19^, ^20^, ^21^, ^22^ However, assessment of the prevalence of heart murmurs in apparently healthy cats by veterinarians in GPs, as described in the present study, has received limited attention^23^ despite the primary role of GPs in identifying heart disease in cats. The results of cardiac auscultation performed by GP veterinarians and by the principal investigator showed minimal agreement, with a kappa value of 0.24. These results indicate that some veterinarians in this study were able to identify heart murmurs and these murmurs tended to be of higher intensity. Results also suggested that GP veterinarians were limited in their ability to detect many heart murmurs. However, such generalizations fail to take into account that systolic heart murmurs in cats often are labile (and the intensity ‐or even presence‐ of a heart murmur at 1 moment in time in a cat does not determine cardiac auscultation findings in the same cat later on), and that some GP veterinarians might have had stronger, or weaker, cardiac auscultatory skills than others.

Other cardiomyopathies were absent from the cats in this study. No cat showed echocardiographic evidence of restrictive, dilated, or arrhythmogenic cardiomyopathy, indicating a prevalence of <0.5% of these disorders in this study sample.

There were several limitations to this study. A significant difference in age existed between groups, and a higher prevalence of HCM exists in older age groups in some case series of cats. This difference could have influenced other variables, although the magnitude of the age difference was small (normal group median age, 7 years [IQR, 4‐10]; abnormal group median age, 9 years [IQR, 7‐11.25]). The lack of male overrepresentation was different from most previous studies.^3^, ^4^, ^12^, ^21^ A possible explanation for both is hidden enrollment bias. For example, GP veterinarians could have selected cats to enroll in the study based on ausculting a heart murmur, which could bias the study sample toward a higher prevalence of heart disease. However, this effect would be expected to be limited, because only 27/217 (12%) of cats in the study had a heart murmur noted at their GPs, and none had a gallop sound or arrhythmia reported by GP veterinarians. This low prevalence is consistent with the recruitment approach, which emphasized contribution to a study and not an appeal for cats suspected of having cardiac disease. Another possible source of error might be misinterpretation of the POC test result. Even though we gave GP veterinarians standard operating procedures, there might still be a possibility that operational error occurred upon performing POC NT‐proBNP ELISA at GPs. We did not request that GP veterinarians take a photo record of each POC test, a step that might be included in future studies to lower operational error on reading the POC result. In the present study, the high level of agreement for POC test results between visual inspection and an automated reader (0/94 cases of discordance when a sample was analyzed with both techniques) suggests that error due to subjectivity on visual inspection of the POC test results was low. A further possible source of error could have been degradation of the NT‐proBNP in vitro. Storage of blood samples for quantitative NT‐proBNP measurement could have allowed temporal degradation of NT‐proBNP and falsely lowered quantitative results. This seems unlikely given the stability of NT‐proBNP for 2 years when frozen at −20°C,^24^ the correlation between quantitative NT‐proBNP and POC NT‐proBNP ELISA, and the fact that there were more samples with high quantitative NT‐proBNP results during the first half of the sampling period (longer storage) than the second half. Another possible source of error could be the imperfection of 2‐dimensional and M‐mode echocardiography for detection of cardiac disease in cats. Echocardiography is the current gold standard test to diagnose feline heart disease, but there are some limitations of this test including intraobserver variation,^16^ the existence of HCM‐associated changes that might not be fully expressed as an HCM phenotype,^25^, ^26^ and that the severity of abnormalities is quantified in different ways. These limitations of echocardiography could all affect the final diagnosis. In this study, all echocardiographic examinations were performed by a single investigator who was blinded to NT‐proBNP test results. This protocol did not formally evaluate intraobserver variation in the performance and interpretation of echocardiograms, however. Finally, the semiquantitative method for categorizing the severity of echocardiographic changes was adapted from a previous study,^12^ but did not undergo prospective validation nor was it based on survival data.

## 5 CONCLUSION

This study showed that POC NT‐proBNP ELISA had low sensitivity and high specificity for detection of heart disease in apparently healthy cats in GPs. While the results suggest that a positive POC NT‐proBNP ELISA test result is associated with cardiac disease, the degree of severity of such disease is variable and an echocardiogram is warranted for cats with a positive POC NT‐proBNP ELISA test result. A negative POC test result is ineffective at ruling out heart disease, such that the POC NT‐proBNP ELISA test should not be used as a screening test. Cardiac auscultation findings affected the utility of POC NT‐proBNP ELISA. Using POC NT‐proBNP ELISA in cats with heart murmurs was associated with a higher sensitivity for identifying structural heart disease than using POC NT‐proBNP ELISA alone.

## CONFLICT OF INTEREST DECLARATION

Etienne Côté and Yong‐Wei Hung have received speaker's honoraria from IDEXX Laboratories in the past 5 years, unrelated to this study. Ta‐Li Lu has received travel and accommodations coverage and speaker's honoraria from IDEXX Laboratories, Inc. Other authors declare no conflicts of interest.

## OFF‐LABEL ANTIMICROBIAL DECLARATION

Authors declare no off‐label use of antimicrobials.

## INSTITUTIONAL ANIMAL CARE AND USE COMMITTEE (IACUC) OR OTHER APPROVAL DECLARATION

Approved by Cardiospecial Veterinary Hospital's Committee on Research Ethics (No. 201701).

## HUMAN ETHICS APPROVAL DECLARATION

Authors declare human ethics approval was not needed for this study.
